# Chromoanasynthetic Genomic Rearrangement Identified in a *N*-Ethyl-*N*-Nitrosourea (ENU) Mutagenesis Screen in *Caenorhabditis elegans*

**DOI:** 10.1534/g3.115.024257

**Published:** 2015-11-30

**Authors:** Omar A. Itani, Stephane Flibotte, Kathleen J. Dumas, Donald G. Moerman, Patrick J. Hu

**Affiliations:** * Institute of Gerontology, University of Michigan Medical School, Ann Arbor, Michigan 48109; † Department of Internal Medicine, University of Michigan Medical School, Ann Arbor, Michigan 48109; ‡ Department of Cell and Developmental Biology, University of Michigan Medical School, Ann Arbor, Michigan 48109; § Department of Zoology, University of British Columbia, Vancouver, British Columbia V6T 1Z3, Canada

**Keywords:** chromoanasynthesis, chromothripsis, *C. elegans*, insulin-like growth factor signaling, dauer

## Abstract

Chromoanasynthesis is a recently discovered phenomenon in humans with congenital diseases that is characterized by complex genomic rearrangements (CGRs) resulting from aberrant repair of catastrophic chromosomal damage. How these CGRs are induced is not known. Here, we describe the structure and function of *dpDp667*, a causative CGR that emerged from a *Caenorhabditis elegans* dauer suppressor screen in which animals were treated with the point mutagen *N*-ethyl-*N*-nitrosourea (ENU). *dpDp667* comprises nearly 3 Mb of sequence on the right arm of the X chromosome, contains three duplications and one triplication, and is devoid of deletions. Sequences from three out of the four breakpoint junctions in *dpDp667* reveal microhomologies that are hallmarks of chromoanasynthetic CGRs. Our findings suggest that environmental insults and physiological processes that cause point mutations may give rise to chromoanasynthetic rearrangements associated with congenital disease. The relatively subtle phenotype of animals harboring *dpDp667* suggests that the prevalence of CGRs in the genomes of mutant and/or phenotypically unremarkable animals may be grossly underestimated.

The incorporation of whole genome sequencing (WGS) into human disease bioanalytic pipelines has led to unexpected insights into the roles of complex genomic rearrangements (CGRs) in the pathogenesis of cancer and congenital disease. The genomes of a significant minority of cancers, and of some patients with congenital abnormalities, harbor CGRs that are thought to result from a catastrophic “chromosome shattering” event known as chromothripsis (Kloosterman *et al.* 2011, 2012; Stephens *et al.* 2011; Zack *et al.* 2013). These rearrangements are surprisingly complex and can result in tumor suppressor inactivation, oncogene amplification, and other abnormalities that may provide cells with a selective advantage during oncogenesis (Molenaar *et al.* 2012; Northcott *et al.* 2012; Rausch *et al.* 2012; Morin *et al.* 2013; Zack *et al.* 2013; Nones *et al.* 2014; George *et al.* 2015). Chromothriptic CGRs can consist of hundreds of rearrangements, are usually localized to one or a few chromosomes, and exhibit copy number changes that alternate between high and low copy number states, with frequent loss of heterozygosity (LOH). Breakpoint junctions in chromothriptic CGRs typically lack homology, suggesting that they are products of nonhomologous end joining (NHEJ). (Kloosterman *et al.* 2011, 2012; Stephens *et al.* 2011; Holland and Cleveland 2012; Kloosterman and Cuppen 2013; Zhang *et al.* 2013; Weckselblatt and Rudd 2015). A similar but distinct class of CGRs identified in patients with congenital developmental disorders is characterized by localized duplications and triplications without LOH. Sequence analysis of these breakpoint junctions reveals short stretches of homology that are signatures of templated DNA repair rather than NHEJ. These CGRs are thought to be the product of a phenomenon distinct from chromothripsis known as chromoanasynthesis (Liu *et al.* 2011; Holland and Cleveland 2012; Kloosterman *et al.* 2012; Kloosterman and Cuppen 2013; Zhang *et al.* 2013; Weckselblatt and Rudd 2015). While catastrophic DNA damage is thought to be a prerequisite for the generation of both classes of CGRs, the inciting events that induce such damage *in vivo* are poorly understood.

Here, we report the analysis of a causative CGR with characteristics of chromoanasynthesis that emerged from a *N*-ethyl-*N*-nitrosourea (ENU)-based genetic screen for suppressors of dauer arrest in the nematode *Caenorhabditis elegans*.

## Materials and Methods

### C. elegans strains and maintenance

Animals were maintained at 15° on nematode growth media (NGM) plates seeded with *Escherichia coli* OP50. Compound mutants were constructed using standard genetic techniques. Genotypes were confirmed by PCR amplification to detect restriction fragment length or PCR polymorphisms. Percival I-36NL incubators (Percival Scientific, Inc., Perry, IA) were used for maintenance and dauer arrest assays. The following mutant alleles were used in this study: *eak-7(tm3188)* (Alam *et al.* 2010), *akt-1(ok525)* (Hertweck *et al.* 2004), and *daf-2(e1368)* (Kimura *et al.* 1997).

### Suppressor of eak-7;akt-1 (seak) screen

The forward genetic screen, WGS, and mapping were performed as previously described (Dumas *et al.* 2013). Animals were exposed to 0.5 mM ENU for 4 hr at room temperature.

### Sequence analysis

Paired-end sequence reads were mapped to the *C. elegans* reference genome version WS230 (www.wormbase.org) using both short-read aligners BWA (Li and Durbin 2009) and Phaster (Philip Green, personal communication). The resulting alignment files were sorted and indexed, and single nucleotide variants (SNVs) were identified with the help of the SAMtools toolbox (Li *et al.* 2009). Copy numbers were estimated in a given genomic interval by dividing the number of aligned reads for strain BQ13 by the number of reads in the corresponding interval for the parental strain, after proper rescaling to a common total number of aligned reads for each library. The estimated copy number in overlapping intervals was examined visually using R (www.r-project.org), and the size of the intervals was varied in order to find the approximate location of each breakpoint. The creation of those overlapping intervals and the calculation of the number of reads within each interval were performed with the BEDTools suite (Quinlan and Hall 2010). Using the IGV genome viewer (Robinson *et al.* 2011; Thorvaldsdottir *et al.* 2013), read alignments around the approximate breakpoints were examined for multiple split-reads with alignments ending at the same location, with the second part of those reads all starting to align at a common location and orientation. In order to confirm the exact breakpoints and junctions found in IGV, a subset of split reads overlapping the junctions were realigned on the reference genome using Blast (Altschul *et al.* 1990) as implemented on WormBase (www.wormbase.org).

### Polymerase chain reaction (PCR)

PCR was performed using Phusion DNA polymerase (New England BioLabs Inc., Ipswich, MA) according to the manufacturer’s protocol. Products were visualized after electrophoresis on a 2% agarose gel containing 0.5 μg/ml ethidium bromide in 1 × Tris-acetate-EDTA (TAE) buffer (primer sequences are provided in Supporting Information, Table S1).

### Dauer arrest assays

Dauer assays were performed at 25° as previously described (Hu *et al.* 2006). Briefly, animals were synchronized in a 4 hr egg-lay at 15° and grown at 25° on NGM plates. Animals were scored ∼60–72 hr after egg-lay. In assays that did not involve RNAi (Figure 5A), animals were fed *E. coli* OP50, and RNAi-based assays (Figure 5B) were conducted using the *E. coli* HT115 feeding RNAi strain (Kamath *et al.* 2001).

## Results and Discussion

In *C. elegans*, a conserved insulin receptor (InsR)/PI 3-kinase/Akt pathway controls larval development and adult life span (Murphy and Hu 2013). Under replete conditions, agonist insulin-like peptides (ILPs) promote reproductive development by activating the InsR ortholog DAF-2, resulting in activation of the serine-threonine kinases AKT-1 and AKT-2, Akt-dependent phosphorylation of the FoxO transcription factor DAF-16, and subsequent inhibition of DAF-16/FoxO through its export from the nucleus and sequestration in the cytoplasm. In unfavorable environments, antagonist ILPs reduce DAF-2/InsR signaling, thus inducing the translocation of unphosphorylated DAF-16/FoxO to the nucleus, where it promotes larval arrest in a state of diapause known as dauer (Murphy and Hu 2013). *daf-2/InsR* loss-of-function mutants undergo dauer arrest constitutively in a *daf-16/FoxO*-dependent manner (Vowels and Thomas 1992; Gottlieb and Ruvkun 1994).

We discovered a conserved protein of unknown function known as EAK-7 that acts in parallel to AKT-1 to inhibit DAF-16/FoxO-dependent dauer arrest and life span extension (Alam *et al.* 2010). In order to identify new DAF-16/FoxO regulators, we performed an ENU mutagenesis screen for suppressors of the dauer-constitutive phenotype of *eak-7;akt-1* double mutants (*seak* mutants). This screen previously revealed a new role for the dosage compensation protein DPY-21 in the regulation of dauer arrest and DAF-16/FoxO activity (Dumas *et al.* 2013).

We subjected all *seak* mutants and the parental *eak-7;akt-1* double mutant strain to WGS. The *seak* phenotype in one mutant strain that emerged from this screen, BQ13, mapped just to the right of an ENU-induced noncoding SNV at ∼11.9 Mb on the reference X chromosome sequence (www.wormbase.org). Among 24 recombinants between this SNV and an ENU-induced SNV in the *rgs-11* gene at ∼14.9 Mb on the right arm of the X chromosome, no recombination was detected between ENU-induced SNVs in the R09A8.2 and *elt-3* genes, located at ∼12.6 Mb and ∼13.9 Mb (corresponding to genetic positions of 7.26 and 15.54 map units), respectively. This observation indicated that the R09A8.2 and *elt-3* SNVs were in linkage disequilibrium and suggested that BQ13 contained a genomic rearrangement in this region that suppresses recombination between R09A8.2 and *elt-3*.

**Figure 1:**
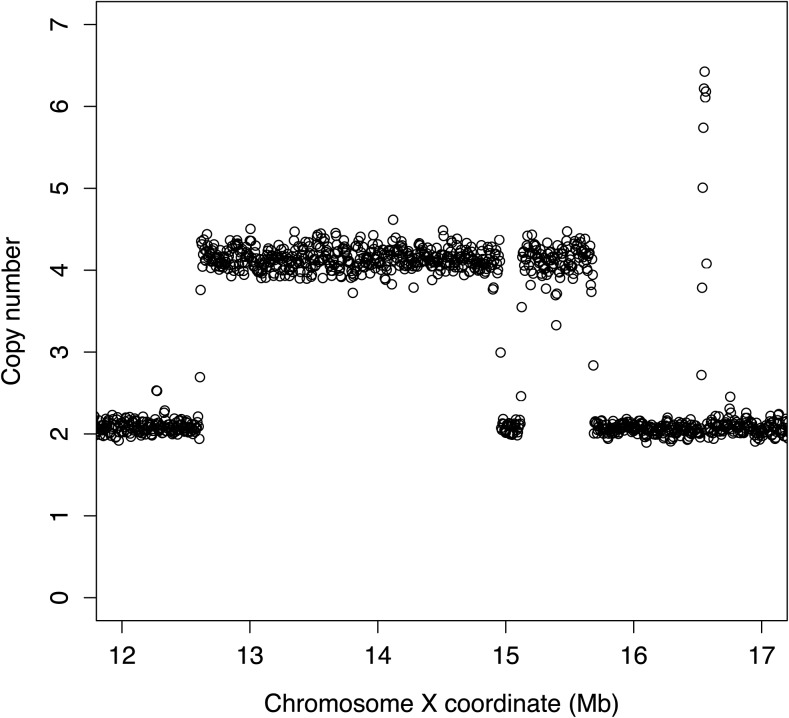
Copy number estimates based on analysis of whole genome sequencing reads from the right arm of the X chromosome. Each circle represents a genomic window of 10 kb. See *Materials and Methods* for details.

**Figure 2:**
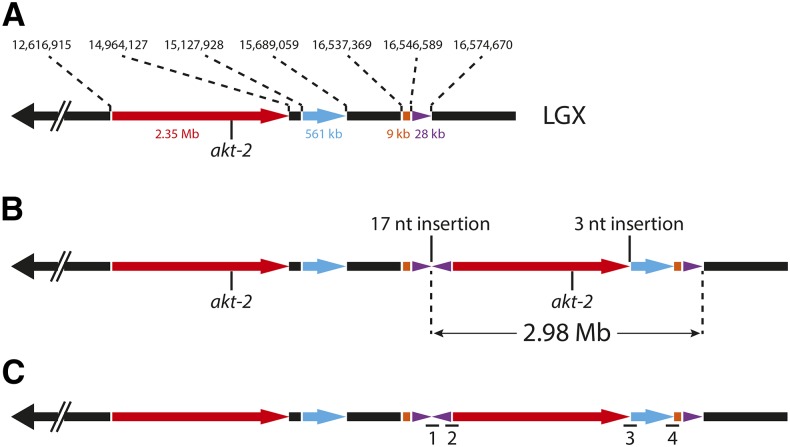
X chromosome schematics. (A) Schematic of the wild-type X chromosome (LGX). Nucleotide coordinates that define boundaries of rearranged genomic segments (colored) and approximate sizes of these segments are denoted above and below the schematic, respectively. 9 and 28 kb segments are not drawn to scale. The location of the *akt-2* gene is shown. (B) Proposed structure of the *dpDp667* CGR (complex genomic rearrangement). Although the inverted segments flanking the 17-nucleotide insertion are depicted as identical, the two segments actually end at slightly different points in the X chromosome sequence. See Figure 4 for details. (C) Location of four breakpoints unique to *dpDp667*. Primers used to amplify breakpoint junction sequences are shown in Table S1, and genomic sequences spanning each breakpoint are shown in Figure 4.

**Figure 3:**
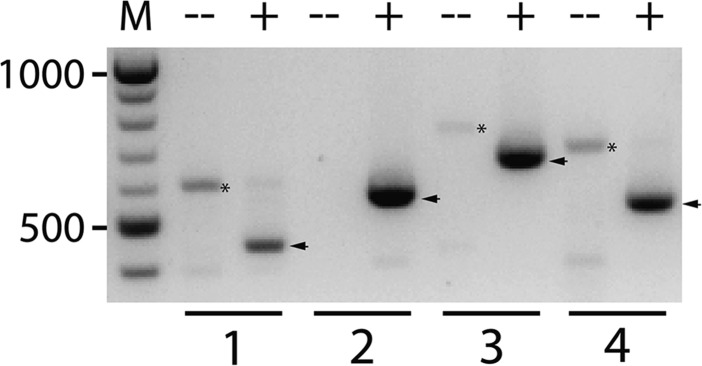
PCR amplification of junction sequences spanning breakpoints 1–4. Predicted sizes of PCR (polymerase chain reaction) fragments are shown in Table S1. For each set of breakpoint junction primers, template DNA isolated from wild-type (−) and *dpDp667* (+) animals was used. Products from *dpDp667* animals (arrows) were extracted from the gel and subjected to Sanger sequencing to verify sequences flanking each breakpoint. (*), nonspecific products amplified from wild-type template; M, molecular weight markers.

**Figure 4:**
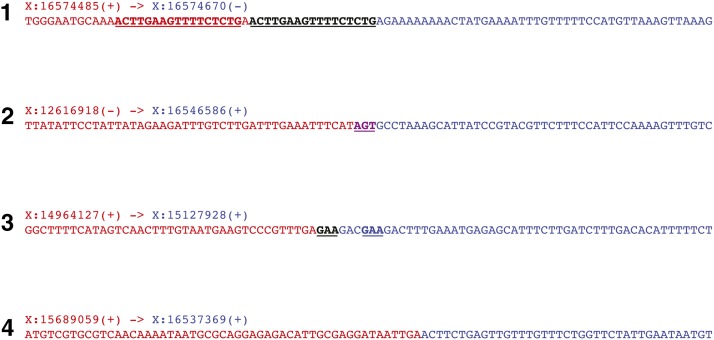
Breakpoint junction sequences. X chromosome coordinates of the nucleotides adjacent to the breakpoint are shown above each sequence. (+): positive DNA strand; (−): negative DNA strand. Junctional insertions in breakpoints 1 and 3 are in black, emboldened, and underlined, and junctional sequences homologous to these insertions are emboldened and underlined. An AGT triplet present at the ends of both fusion partners of breakpoint junction 2 is in purple, emboldened, and underlined. See text for details.

Analysis of sequencing read depth from the right arm of the X chromosome in the BQ13 sample revealed two regions of copy number doubling spanning approximately 2 Mb and 500 kb, and one smaller region of apparent copy number tripling (Figure 1). Inspection of individual reads revealed four distinct breakpoint junctions in BQ13, each of which was identified in multiple reads. None of these hybrid reads was present in the parental *eak-7;akt-1* samples. These data were consistent with the presence of a CGR on the right arm of the X chromosome (Figure 2). To verify the proposed structure of this CGR, PCR primers were designed to amplify each of the four predicted breakpoint junctions in BQ13 (Figure 2C and Table S1). Each primer set amplified a fragment of the predicted size (Table S1) from BQ13 genomic DNA but not from wild-type genomic DNA (Figure 3), and Sanger sequencing of these PCR products verified breakpoint sequences identified in WGS reads (Figure 4). In accordance with conventions of *C. elegans* nomenclature [(Horvitz *et al.* 1979); Tim Schedl, personal communication], we refer to this CGR as *dpDp667*, since the rearrangement consists mostly of duplicated sequence.

*dpDp667* has two main features that suggest it is a product of chromoanasynthesis rather than chromothripsis. First, it contains three duplications and a triplication but does not harbor any deletions. This contrasts with the typical alternation of high and low copy number states that is seen in chromothripsis (Stephens *et al.* 2011; Holland and Cleveland 2012; Kloosterman and Cuppen 2013; Zhang *et al.* 2013; Weckselblatt and Rudd 2015). Furthermore, breakpoint junctions 1 and 3 contain short insertions that are homologous to nearby junctional sequences, and junction 2 contains a triplet that is present at both ends of the breakpoint (Figure 4). These findings suggest that these three fusions are products of templated DNA repair, which is a characteristic of chromoanasynthetic CGRs (Liu *et al.* 2011; Holland and Cleveland 2012; Kloosterman and Cuppen 2013; Zhang *et al.* 2013; Weckselblatt and Rudd 2015). It is conceivable that the cellular machinery responsible for generating chromoanasynthetic CGRs in the germline is conserved between humans and *C. elegans*.

Due to their complexity, the functional significance of most reported CGRs has not been ascertained experimentally. In a previous study, we showed that mutations in *dpy-21*, the first gene to emerge from our *seak* screen, suppress the dauer-constitutive phenotypes of *eak-7;akt-1* double mutants and *daf-2/InsR* mutants at least in part by increasing *akt-2* expression (Dumas *et al.* 2013). Since the *akt-2* gene is duplicated in *dpDp667* (Figure 2), we hypothesized that *dpDp667* suppresses dauer arrest by increasing *akt-2* gene dosage.

**Figure 5:**
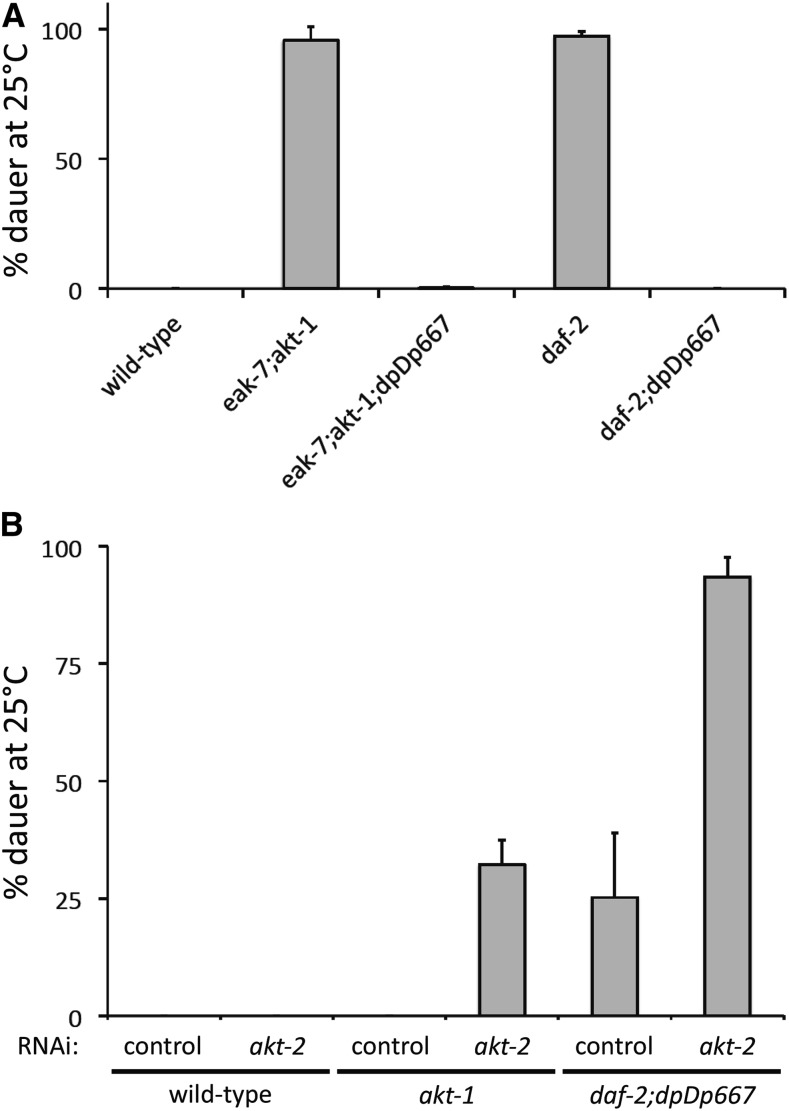
*dpDp667* suppresses dauer arrest in an *akt-2*-dependent manner. (A) *dpDp667* suppresses the dauer-constitutive phenotype of *eak-7;akt-1* double mutants and *daf-2* single mutants. (B) Suppression of the dauer-constitutive phenotype of *daf-2* mutants by *dpDp667* requires *akt-2*. See text for details. Both panels show composite results from three independent trials. Error bars represent standard deviation. RNAi, RNA interference.

As AKT-2 acts in the DAF-2/InsR pathway to prevent dauer arrest and promote reproductive development (Paradis and Ruvkun 1998), we first tested the ability of *dpDp667* to suppress the dauer-constitutive phenotype of the *daf-2(e1368)* mutant (Kimura *et al.* 1997). *dpDp667* suppressed the dauer-constitutive phenotypes of both *daf-2(e1368)* mutants as well as *eak-7;akt-1* double mutants (Figure 5A). We then directly tested the role of *akt-2* in dauer suppression by *dpDp667*. If *dpDp667* suppresses dauer arrest by increasing *akt-2* gene dosage, then RNAi knockdown of *akt-2* should increase the penetrance of dauer arrest in *daf-2;dpDp667* animals. *akt-2* RNAi did not induce dauer arrest at 25° in wild-type animals but did cause partially penetrant dauer arrest in *akt-1* mutant animals (Figure 5B), indicating that *akt-2* RNAi reduced *akt-2* activity (Paradis and Ruvkun 1998). In contrast to complete suppression of the *daf-2(e1368)* dauer-constitutive phenotype by *dpDp667* observed when grown on the standard *E. coli* OP50 strain, *daf-2;dpDp667* animals had a partially penetrant dauer-constitutive phenotype when grown on the HT115 feeding RNAi strain (compare Figure 5, A and B). This is likely a consequence of the influence of *E. coli* strain-specific differences on dauer arrest (Ferguson *et al.* 2013). *akt-2* RNAi strongly enhanced the penetrance of dauer arrest in *daf-2;dpDp667* animals (Figure 5B). Therefore, we conclude that *dpDp667* suppresses the dauer-constitutive phenotype of *daf-2/InsR* mutants at least in part through increased *akt-2* gene dosage. We cannot exclude the possibility that increased dosage of other genes in *dpDp667* also contributes to the dauer suppression phenotype.

Although progress has been made in understanding the biogenesis of CGRs (Crasta *et al.* 2012; Zhang *et al.* 2015), the initial events leading to chromothripsis and chromoanasynthesis are poorly understood. Recent work in *C. elegans* suggests that CGRs can be induced in wild-type animals by the alkylating agent mechlorethamine (Meier *et al.* 2014). Our discovery of a causative CGR in a ENU-based genetic screen was unexpected, given the propensity of ENU to cause point mutations (Flibotte *et al.* 2010). This finding suggests that, in principle, CGRs could arise from any physiological or pathological process that causes point mutations. Since ENU can induce large copy-number duplications/deletions at low frequency *in vivo* [∼0.3%; (Thompson *et al.* 2013)], an alternative model is that *dpDp667* arose from a rare double-strand break induced by ENU. We also cannot exclude the possibility that this CGR arose spontaneously, albeit in the context of a genetic screen.

The discovery of chromothripsis and chromoanasynthesis was a direct consequence of the use of WGS to analyze the genomes of cancer cells (Stephens *et al.* 2011) and patients with congenital developmental abnormalities (Kloosterman *et al.* 2011; Liu *et al.* 2011). Here, we report the first detailed structure of a chromoanasynthetic rearrangement in *C. elegans* that causes a mutant phenotype. The structures of CGRs in *C. elegans* had not been reported prior to the advent of WGS. This is likely due to difficulties in resolving the structures of CGRs in the absence of WGS data ,as well as the known bias of commonly used mutagens such as ENU and ethyl methanesulfonate (EMS) toward point mutations (Flibotte *et al.* 2010). In line with the mutagenic spectrum of EMS and ENU, WGS analytic pipelines are typically optimized for the identification of SNVs (Doitsidou *et al.* 2010; Zuryn *et al.* 2010).

As the BQ13 mutant strain that emerged from our screen does not appear to have gross phenotypic abnormalities, it is likely that other mutants that have been isolated from forward genetic screens but have not been subjected to WGS may harbor cryptic causative CGRs that are well tolerated by the organism. Such CGRs have already been documented in humans (Bertelsen *et al.* 2015; de Pagter *et al.* 2015). Furthermore, it is possible that the structure of previously described simple rearrangements, most of which have not been analyzed using WGS, may be more complex than previously appreciated. In all of these situations, analysis of read depth (Figure 1) would likely reveal evidence of a cryptic CGR. Therefore, we advocate the routine incorporation of read depth analysis into WGS pipelines. As WGS has now become a standard tool in the analysis of mutant genomes (Hu 2014), we anticipate the discovery of many more CGRs as the underlying cause of mutant phenotypes in *C. elegans* and other organisms.

## Supplementary Material

Supporting Information
