# Supplementary material for: Chromoanasynthetic Genomic Rearrangement Identified in a N-Ethyl-N-Nitrosourea (ENU) Mutagenesis Screen in Caenorhabditis elegans
Source: G3 (Bethesda). 2015 Nov 30;6(2):351–6. doi: 10.1534/g3.115.024257 (PMC4751554; doi:10.1534/g3.115.024257)
Supplement: Supporting Information [file supp_6_2_351__index.html]

Chromoanasynthetic Genomic Rearrangement Identified in a N-Ethyl-N-Nitrosourea (ENU) Mutagenesis Screen in Caenorhabditis elegans — Supporting Information 

# Chromoanasynthetic Genomic Rearrangement Identified in a *N*-Ethyl-*N*-Nitrosourea (ENU) Mutagenesis Screen in *Caenorhabditis elegans*

## Supporting Information for Itani *et al.*, 2016

**Files in this Data Supplement:**

- Table S1 - Primers used to amplify four breakpoint junctions in strain BQ13. (.pdf, 22 KB)
