# Supplementary material for: Chromoanasynthetic Genomic Rearrangement Identified in a N-Ethyl-N-Nitrosourea (ENU) Mutagenesis Screen in Caenorhabditis elegans
Source: G3 (Bethesda). 2015 Nov 30;6(2):351–6. doi: 10.1534/g3.115.024257 (PMC4751554; doi:10.1534/g3.115.024257)
Supplement: Supporting Information [file supp_g3.115.024257_TableS1.pdf]

Table S1. Primers used to amplify four breakpoint junctions in strain BQ13. Chromosomal locations are based on the *C. elegans* reference genome version WS230 (www.wormbase.org).

Breakpoint junction	PCR product (bp)	Forward primer	Location on LGX	Reverse primer	Location on LGX
1	460	tgttttcggagcaacttggg	16,574,592-16,574,611	tcacgagatctatcaatgcact	16,574,268-16,574,289
2	584	atcttctgccttggcgagat	16,547,022-16,547,003	cacttggactctctgggtgt	12,617,205-12,617,186
3	688	cccctctacttggaccttgt	14,963,918-14,963,937	ggggcagcaacctctttcta	15,128,402-15,128,383
4	553	gcgcacccctgattcctatt	15,688,954-15,688,973	agctcgaccatttgcattgg	16,537,820-16,537,801
